# Evidence That Multiple Defects in Lipid Regulation Occur before Hyperglycemia during the Prodrome of Type-2 Diabetes

**DOI:** 10.1371/journal.pone.0103217

**Published:** 2014-09-03

**Authors:** Simon G. Anderson, Warwick B. Dunn, Moulinath Banerjee, Marie Brown, David I. Broadhurst, Royston Goodacre, Garth J. S. Cooper, Douglas B. Kell, J. Kennedy Cruickshank

**Affiliations:** 1 Institute of Cardiovascular Sciences, Core Technology Facility, The University of Manchester, Manchester, United Kingdom; 2 Manchester Centre for Integrative Systems Biology, Manchester Institute of Biotechnology, The University of Manchester, Manchester, United Kingdom; 3 Centre for Advanced Discovery & Experimental Therapeutics (CADET), Central Manchester NHS Foundation Trust and School of Biomedicine, The University of Manchester, Manchester Academic Health Sciences Centre, Manchester, United Kingdom; 4 School of Biosciences, University of Birmingham, Edgbaston, Birmingham, United Kingdom; 5 Division of General Internal Medicine, Department of Medicine, 4126A Katz Group Centre for Pharmacy & Health, University of Alberta, Edmonton, Alberta, Canada; 6 Maurice Wilkins Centre for Molecular Biodiscovery, Faculty of Science, University of Auckland, Auckland, New Zealand; 7 Department of Pharmacology, University of Oxford, Oxford, United Kingdom; 8 Diabetes & Nutritional Sciences Division, King's College London, London, United Kingdom; Virgen Macarena University Hospital, School of Medicine, University of Sevillem, Spain

## Abstract

**Background:**

Blood-vessel dysfunction arises before overt hyperglycemia in type-2 diabetes (T2DM). We hypothesised that a metabolomic approach might identify metabolites/pathways perturbed in this pre-hyperglycemic phase. To test this hypothesis and for specific metabolite hypothesis generation, serum metabolic profiling was performed in young women at increased, intermediate and low risk of subsequent T2DM.

**Methods:**

Participants were stratified by glucose tolerance during a previous index pregnancy into three risk-groups: overt gestational diabetes (GDM; *n* = 18); those with glucose values in the upper quartile but below GDM levels (UQ group; *n* = 45); and controls (*n* = 43, below the median glucose values). Follow-up serum samples were collected at a mean 22 months postnatally. Samples were analysed in a random order using Ultra Performance Liquid Chromatography coupled to an electrospray hybrid LTQ-Orbitrap mass spectrometer. Statistical analysis included principal component (PCA) and multivariate methods.

**Findings:**

Significant between-group differences were observed at follow-up in waist circumference (86, 95%CI (79–91) vs 80 (76–84) cm for GDM vs controls, p<0.05), adiponectin (about 33% lower in GDM group, p = 0.004), fasting glucose, post-prandial glucose and HbA_1c_, but the latter 3 all remained within the ‘normal’ range. Substantial differences in metabolite profiles were apparent between **the 2 ‘at-risk’** groups **and controls**, particularly in concentrations of phospholipids (4 metabolites with p≤0.01), acylcarnitines (3 with p≤0.02), short- and long-chain fatty acids (3 with p< = 0.03), and diglycerides (4 with p≤0.05).

**Interpretation:**

Defects in adipocyte function from excess energy storage as relatively hypoxic visceral and hepatic fat, and impaired mitochondrial fatty acid oxidation may initiate the observed perturbations in lipid metabolism. Together with evidence from the failure of glucose-directed treatments to improve cardiovascular outcomes, these data and those of others indicate that a new, quite different definition of type-2 diabetes is required. This definition would incorporate disturbed lipid metabolism prior to hyperglycemia.

## Introduction

The metabolic basis of type 2 diabetes mellitus (T2DM) has traditionally had hyperglycemia as its *sine qua non*, despite generally being accompanied by a long prior history of (central) obesity together with relative physical inactivity. Evidence suggests that blood vessel dysfunction, either overt or inducible, is detectable prior to rises in blood glucose [1]–[3], as occurs in the disease itself [4]. Debate over whether glucose is the direct cause of the blood vessel damage has not yet been resolved. Many lines of evidence suggest that hyperglycemia may not be the earliest metabolic change in the complications of T2DM. One, based on current treatment results in clinical trials, is that complications are not prevented by glycemic control, intensive or not [5]–[7], confirmed by the latest very large trials of dipeptidyl peptidase-4 (DPP-4) inhibitors [8]. Earlier evidence suggested that microvascular components were delayed more by lowered blood pressure [6], [9]–[11] than by tight blood glucose control. The ACCORD (Action to Control Cardiovascular Risk in Diabetes) trial reported no overall difference in microvascular outcomes in diabetic subjects at risk of cardiovascular events, and intensive glycemic treatment was associated with higher mortality [12]. HMG CoA reductase inhibitors with its anti-flammatory and anti-thrombotic effects [13], [14], have been used to target successfully total and LDL-cholesterol [15], [16]. Despite such reductions, intriguingly statin treatment may marginally increase glycemia [17]–[19]. A second line of evidence is that the impaired blood vessel responsiveness is in both large arteries *in vivo* [1] and smaller arteries in tissue biopsy studies, which occurs even when blood glucose is normal [20]. A third set of arguments, coupled to an extensive literature reviewed elsewhere, is that iron and copper dysregulation are implicated in diseases that manifest in changes in both lipid and carbohydrate metabolism (and their attendant co-morbidities) [21], [22]

Metabolomics is a systems biology strategy for exploring the low molecular weight metabolites present in the metabolome of an organism [23]. It portrays a dynamic interaction of a phenotype with the environment, across genomic and post-transcriptional regulation [23] and has been applied to study cardiovascular diseases [24]–[29] including heart failure [30], myocardial ischemia [31], [32], myocardial infarction [33] and preeclampsia [34]. Its application in the investigation of glucose intolerance [35]–[38] has led to the identification of new metabolic biomarkers and has highlighted the influence of drugs on the metabolic profile of subjects diagnosed with glucoregulatory disorders [39], [40]. Animal studies using targeted metabolomic approaches have confirmed that mitochondrial overload and incomplete fatty acid oxidation in skeletal muscle occur in both major types of diabetes [41].

Gestational glycemic status including overt gestational diabetes mellitus (GDM) increases susceptibility to subsequent development of the T2DM ‘phenotype’ postnatally [42], although a confounding factor is obesity [43].

Here, we examined the early metabolic natural history of ‘pre-diabetes’ by comparing the serum metabolic profiles of women from three backgrounds, systematically determined in the third trimester of pregnancy. However, here, we chose a data-driven approach free of specific hypotheses [44] to determine which metabolite classes might be so changed on a number of pathways. All these women were followed for some two years postnatally when serum samples for metabolomic analysis were taken. Our main hypothesis was that the metabolome at follow-up would differ significantly between those women at high risk of T2DM (having had previous GDM) compared to those who remained normoglycemic throughout pregnancy and a third group who were normoglycemic during pregnancy but in the upper quartile of the glycemic distribution. Samples of these women were included in the vascular sub-study [3].

## Research Design and Methods

### Ethics statement

All protocols were approved by the Central Manchester Local Research Ethics Committee (LREC No. 03/CM/477: Approval date 15 June 2004). Participants were fully informed about the nature, goal, procedures and risks of the study, and gave their informed consent in writing.

### Study Population

The Hyperglycemia and Pregnancy Outcome (HAPO) study was a multi-centre study investigating the impact of glycemia below (but not including) overt diabetes in singleton pregnancies of women not taking anti-hypertensive drugs nor any other chronic therapies. Inclusion criteria were that women were at least 3 months pregnant, were to deliver at our local maternity hospital, and had completed a 75 g oral glucose tolerance test (GTT) at 24–32 weeks gestation.

**Figure 1:**
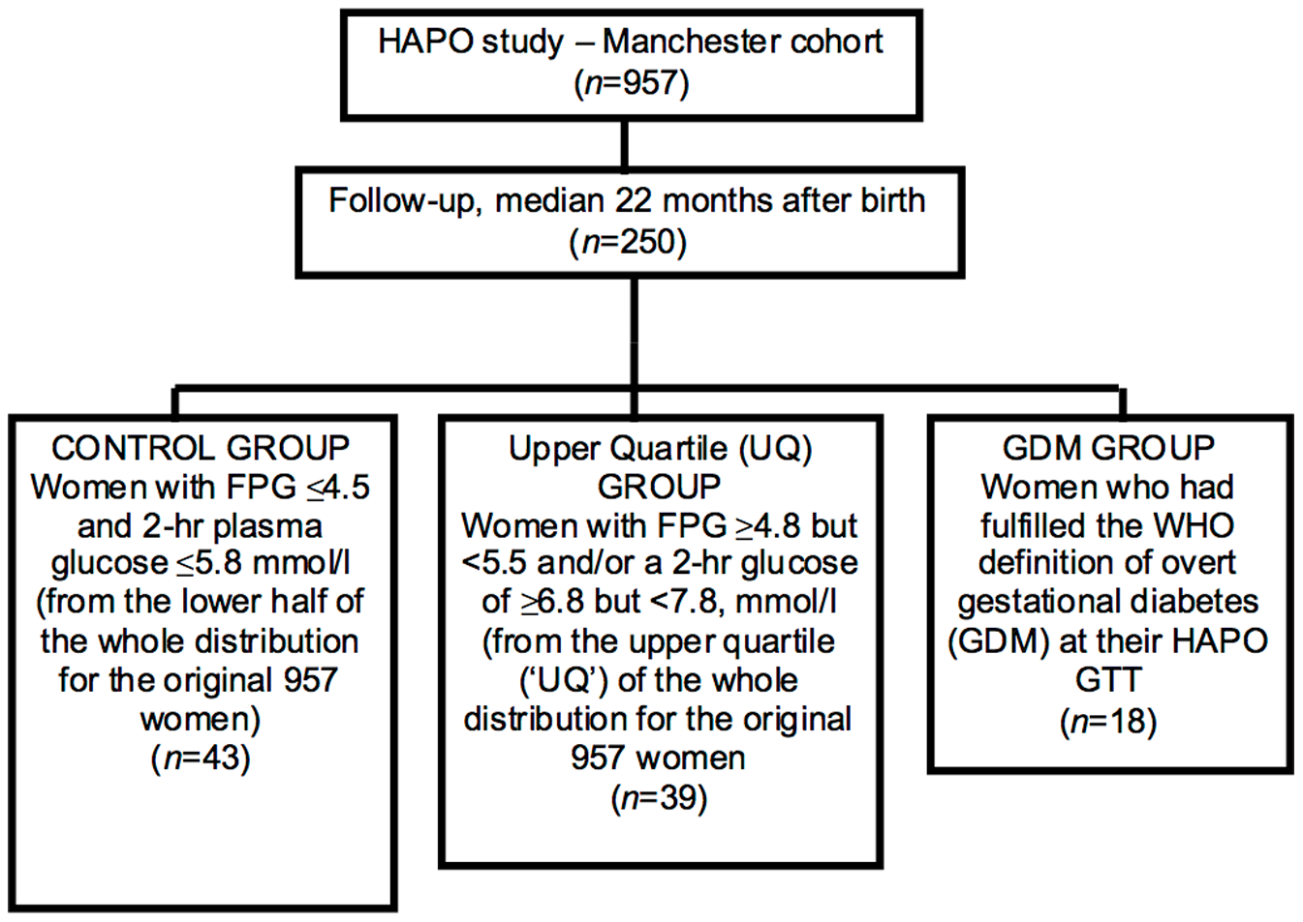
The sample selection process.

To establish our sampling frame (Figure 1), we used the glycemic distribution from the first 957 participants recruited at the Manchester site of the Hyperglycemia and Pregnancy Outcome (HAPO) study [45]. From the group of 250 of these women who were initially followed up, we selected 100 women, including all 18 with previous GDM and 82 additional participants by computer-generated random sampling, who were stratified into the upper quartile of the original glycaemic distribution (UQ group) or below that distribution's median (control group). They were then matched for confounding factors of age, BMI and ethnicity in that order. There was no prior nor current use of statins/other cardiometabolic medications in these young women. The three final study groups were as follows: i) the 18 women who fulfilled the WHO definition of overt GDM at their HAPO GTT (GDM group); ii) 39 women with an index gestational fasting plasma glucose (FPG) value ≥4.8 but <5.5 mmol·L^−1^, and/or a 2 hr glucose value of ≥6.8 but <7.8, mmol·L^−1^ (i.e. these were the upper quartile (‘UQ’) cut-off values for the whole distribution in the 957 original women, below GDM, forming the UQ group); and iii) 43 women whose gestational FPG had been ≤4.5, and 2-hr plasma glucose ≤5.8, mmol·L^−1^ (from the lower half of the original GTT distribution) (Control Group) – see Figure 1. Follow-up was performed at a mean of 22 months after the index pregnancy when fasting blood serum samples for metabolic profiling and, if possible, 2-h GTTs were, repeated.

### Anthropometric measurements

All anthropometric measurements were taken by trained staff following WHO guidelines [46]. Total body-fat estimation was via a widely employed bioimpedance method (Bodystat 1500, Bodystat Ltd, UK).

### Biochemical measurements

Blood samples were centrifuged, and serum and plasma aliquotted, immediately frozen and maintained at −80°C for later analysis of lipids and hormones. Blood samples were analyzed for glucose, triglyceride (TG), total cholesterol (TC), high-density lipoprotein cholesterol (HDL-C), low-density lipoprotein cholesterol (LDL-C), NEFA, insulin, adiponectin, and leptin. We measured glucose by the glucose oxidase method on a Beckman Synchron LX system. HbA_1c_ was assayed by reversed phase cation exchange chromatography (Menarini Diagnostic, UK). Serum adiponectin and leptin were measured using ELISA (R&D Systems, Minneapolis, MN), and insulin with monoclonal-based ELISAs (Mercodia, Sweden). Serum TC and TG were measured by the CHOD/PAP and GPO/PAP methods respectively on a Cobas Mira S analyzer (ABX Diagnostics, Shefford, UK); all reagents were obtained from the same source. HDL-C was measured by a second-generation homogenous method using PEG-modified enzymes (Roche Diagnostics, Lewes, UK). LDL-C was calculated using the Friedewald formula. A calculated LDL-C value of <0.1 mmol.L^−1^ was set as the detection limit for cholesterol. Finally, we measured non-esterified free fatty acids (NEFA) in plasma using an enzymatic endpoint assay (WAKO Chemicals, Richmond VA) with a detection limit of 0.01 mmol.L^−1^.

### Preparation of serum samples for metabolomic analysis

Fasting serum samples taken at follow-up, with group of origin blinded to the analyst, were thawed on ice and prepared as previously described [47], [48]. Samples were deproteinised by mixing 200-µL plasma with 600-µL methanol followed by vortex-mixing (15 s) and centrifugation (15 min, 13,865 *g*). 370-µL aliquots of each supernatant were transferred to two Eppendorf tubes and lyophilised (HETO VR MAXI vacuum centrifuge attached to a Thermo Svart RVT 4104 refrigerated vapour trap; Thermo Life Sciences, Basingstoke, UK). The aliquots were for separate positive- and negative-ion Ultra Performance Liquid Chromatography-Mass Spectrometry (UPLC-MS) analyses, respectively. A pooled quality control (QC) sample [47] was also prepared by mixing 50-µL aliquots of serum from each of 100 subject samples followed by vortex mixing for one minute. 200-µL aliquots of the pooled QC sample were deproteinised and lyophilised as described above.

### UPLC-MS analysis

Each sample was reconstituted in 100-µL water and vortex mixed (15 s) and then centrifuged (15 min, 13,865 *g*). Samples were analysed in a random order using Ultra Performance Liquid Chromatography (UPLC; Waters, Elstree, UK) coupled to an electrospray hybrid LTQ-Orbitrap mass spectrometer (ThermoFisher Scientific, Bremen, Germany). Each sample was analysed twice, once in negative-ion mode and once in positive-ion mode. The analytical conditions [47], [49] and application of QC samples [47] were applied as described previously.

### Raw data processing and data analysis

Raw data files (.RAW) were converted to the NetCDF format using the File converter program in XCalibur (ThermoFisher Scientific, Bremen, Germany). Deconvolution of data was performed using XCMS as described previously [48], as were signal correction and quality assurance procedures [34].

### Statistical analysis

Statistical analyses were carried out using STATA version 12 (Stata Corporation, College Station, Texas) or programs written in the Matlab® scripting language (version 7.8; http://www.mathworks.com/). Summary statistics of non-normally distributed continuous variables are presented as geometric means – derived from log-transformed data. Univariate analysis was performed using the Mann-Whitney U test, a non-parametric method for assessing whether two independent samples come from the same distribution. We used maximum-likelihood multinomial logit models to assess the relationship between levels of adiponectin, leptin, indices of adiposity (BMI), smoking status, triglyceride, non-esterified fatty acids (NEFA) as well as cholesterol and the likelihood of having GDM or the UQ of glycemia compared to the control group. Missing values were ignored.

### Annotation of putative metabolites matched to features

Metabolic features characterized by measuring both the accurate *m/z* and retention time, and corresponding putative molecular annotations were assigned by standard methods as described [50]. One or more molecular formulae within available databases were assigned to each feature with mass accuracy of ±3 ppm. These were subsequently searched against The Manchester Metabolomics Database, which has been constructed with information from the Human Metabolome Database (http://www.hmdb.ca/, v2.0) and Lipidmaps (http://www.lipidmaps.org/). This is a level 2 annotation according to the proposed reporting standards of the Metabolomics Standards Initiative [51]. In these types of raw metabolomic data, a single metabolic feature can be assigned to one or more metabolites due to uncertainty caused by possible isomerism, resulting in a non-specific annotation. A higher confidence of a unique annotation can be performed, where experimentally feasible, if the accurate mass, collision-induced dissociation mass spectra and retention time are matched with that of an authentic chemical standard analysed under identical analytical conditions. This is considered to be a level 1 identification according to the reporting standards defined by the Metabolomics Standards Initiative [51]. Where more than one putative structure can be assigned to any analytical feature corresponding to a particular molecular mass (that is, more than one molecule of the particular mass could occur in physiology), each possible annotation has been listed with ‘AND/OR’ as the conjunction. To minimise the influence of false discovery we grouped metabolites based on biological function or chemical structure. The relative ‘hierarchies by *p value*’ are shown in the Result Tables. We performed univariate as well as unsupervised multivariate analyses using principal component analyses (PCA). PCA showed no clustering related to class or sub-clustering of subjects from one or multiple classes therefore these data were not included in the manuscript. Similarly, Partial Least Squares – Discriminant analyses (PLS-DA) was also performed but no validated models were constructed and therefore these data were not reported.

## Results

### Subject group characteristics

Standard anthropometric and metabolic parameters were measured in all participants, and stratified by glycemic status (Table 1). No significant between-group differences were present in age, ethnicity, BMI nor smoking status at follow-up. Small but significant differences in fasting and two-hour serum glucose concentrations occurred during pregnancy as expected. Significant between-group differences were observed at follow-up in waist circumference, adiponectin, fasting glucose, post-prandial glucose and HbA_1c_, with means and all ranges are still within the ‘normal’ range (as defined by WHO), between control and both UQ and GDM women.

**Table 1:** Clinical data for participants during pregnancy and at follow-up in the three study groups.

	Control	UQ	GDM	F and p values
N	43	39	18	
**Mean (95% CI)**	**During Pregnancy**			
*Fasting glucose (mmol.L-1)	4.2 (4.1, 4.3)	4.9 (4.8, 5.0)	4.7 (4.5, 4.8)	F = 36.28; *p*<0.0001
*Two-hour glucose (mmol.L-1)	4.9 (4.7, 5.2)	6.6 (6.3, 6.8)	9.2 (8.9, 9.6)	F = 188.29; *p*<0.0001
	**At Follow-up**			
Age (years)	34.9 (33.5, 36.4)	35.6 (34.0, 37.2)	37.1 (34.8, 39.5)	NS
Ethnicity *n* (%)				
European	32 (44.4)	29 (40.3)	11 (15.3)	
SA	8 (38.1)	6 (28.6)	7 (33.3)	NS**
Others	3 (40.0)	4 (60.0)	0 (0)	
BMI (kg/m2)	25.3 (23.5, 27.1)	27.6 (25.7, 29.6)	27.6 (24.8, 30.5)	NS
Smoking Status (*n*)				
Never	31	22	14	
Ex	5	12	2	NS**
Current	7	5	2	
Day of cycle (median, IQR)	14 (5–21)	13 (10–15)	19 (14–28)	NS
Oral contraceptive use (n)	10	8	5	NS**
Waist circumference (cm)	80 (76, 84)	87 (83, 91)	86 (79, 91)	F = 3.22; *p* = 0.044
Fat (%)	33.5 (31.4, 35.7)	36.5 (34.2, 38.7)	36.5 (33.1, 39.9)	NS
HbA1c	4.8 (4.7, 4.9)	5.0 (4.9, 5.1)	5.1 (4.9, 5.2)	F = 5.75; *p* = 0.004
Fasting Glucose (mmol.L–1)	4.8 (4.6, 4.9)	5.0 (4.8, 5.1)	5.1 (4.8, 5.3)	F = 3.61; *p* = 0.031
2h GTT glucose (mmol.L-1)	5.4 (4.8, 5.9)	6.2 (5.6, 6.8)	7.3 (6.5, 8.1)	F = 7.83; p = 0.001
NEFA (mmol.L-1)	0.19 (0.16, 0.23)	0.21 (0.17, 0.24)	0.15 (0.10, 0.21)	NS
Total Cholesterol (mmol.L-1)	4.4 (4.1, 4.6)	4.2 (4.0, 4.6)	4.2 (3.8, 4.6)	NS
LDL-C (mmol.L-1)	2.5 (2.3, 2.8)	2.5 (2.2, 2.7)	2.5 (2.1, 2.9)	NS
HDL-C (mmol.L-1)	1.46 (1.34, 1.59)	1.35 (1.22, 1.47)	1.34 (1.15, 1.52)	NS
Triglycerides (mmol.L-1)	0.8 (0.7, 0.9)	1.0 (0.9, 1.1)	0.9 (0.7, 1.1)	NS
#Fasting Insulin (pmol/L)	6.1 (4.9, 7.7)	5.7 (4.5, 7.2)	6.9 (4.9, 9.7)	NS
#Adiponectin (mg/L)	3.5 (3.0, 4.1)	3.0 (2.6, 3.5)	2.3 (1.9, )	F = 5.78; p = 0.004
Leptin (ng/mL)	19.0 (13.6, 24.5)	25.4 (19.8, 31.1)	22.9 (14.7, 31.1)	NS

*p* values calculated applying ANOVA or **Chi-squared tests. #Data are geometric mean and 95% confidence intervals

In maximum-likelihood multinomial logit models, increasing adiponectin concentrations (60% reduction in risk per mg.L^−1^) was independently associated with a GDM classification compared to control (Relative risk ratios: 0.41 (0.22, 0.78), p = 0.005) in a model including age (1.16 (1.00, 1.37)), BMI (1.03 (0.86, 1.24)), history of smoking (0.75 (0.93, 1.99)), Ln NEFA (0.85 (0.29, 2.50)), total cholesterol (0.87 (.38, 1.99)), leptin (0.99 (0.93, 1.07) and triglycerides (0.49 (0.11, 2.18)).

### Between-group differences in metabolite concentrations

**Figure 2:**
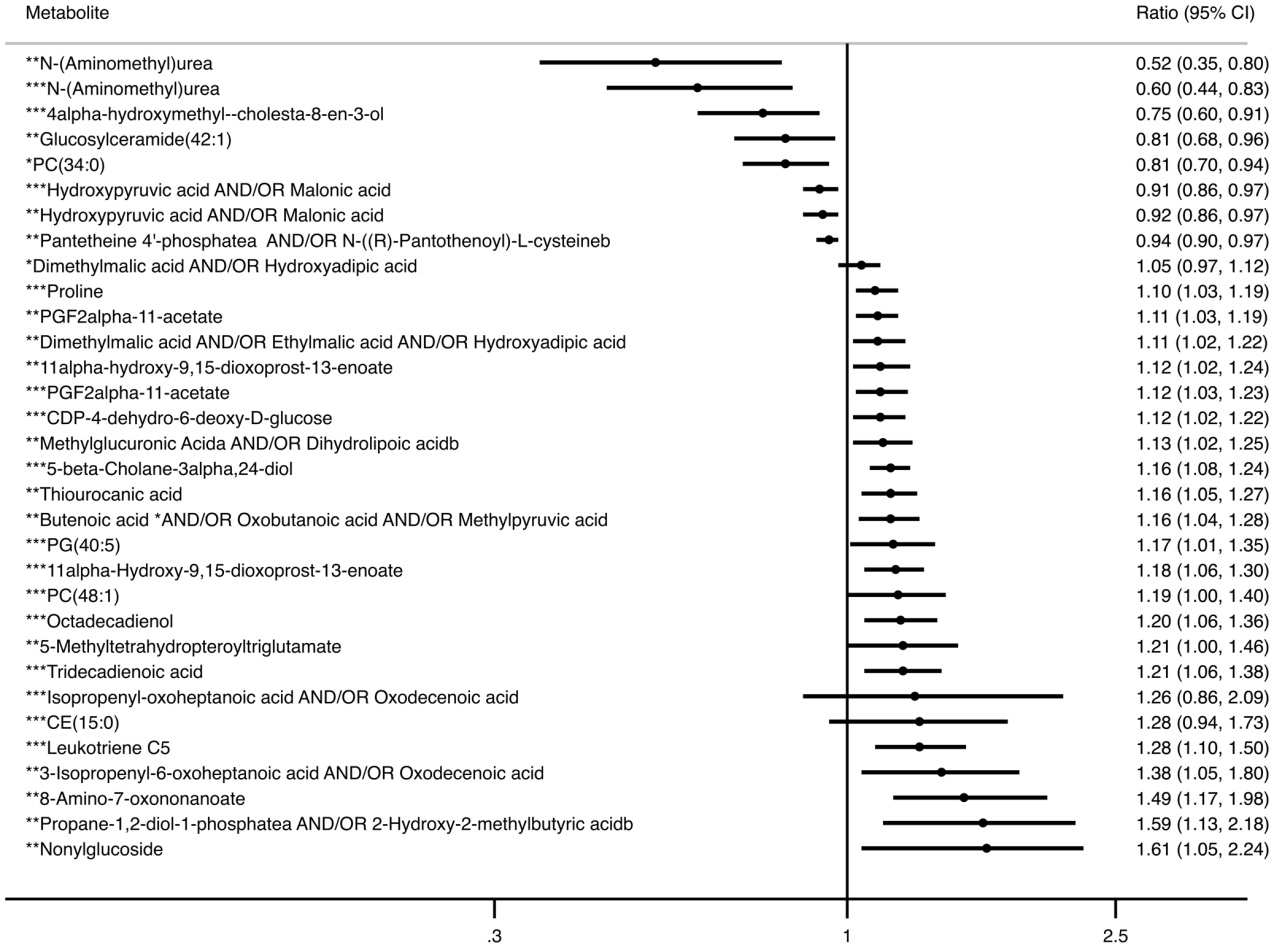
Fold differences metabolites for top 32 metabolites with lowest p values at 2-y follow-up. Metabolites have been classified into classes (*control vs UQ, **control vs GDM and ***UQ vs GDM). Data are in ascending order (lowest to those with the highest) of the ratio of difference between groups. All have *p* values of <0.01.

3,552 metabolomic features were judged suitable for univariate analysis after raw metabolite data and related quality assurance processes had been performed. Levels of numerous metabolites differed significantly between groups. Data are presented (Tables 2 to 4), according to a metabolite classification system in which each molecule is listed as a member either of a *structural* class (e.g. ‘short-chain fatty acids and related metabolites’) or a *functional* class according to its participation in a defined metabolic process (e.g. <participating in> ‘tetrahydrofolate metabolism’). Each metabolite has been listed only once as a member of a single class. If a metabolite was detected more than once, the feature with the lowest *p* value was reported. Within each class, data have been separated into those with higher and lower ratios and are then presented in order from lowest to highest *p*-value. Figure 2 shows in ascending order of fold difference, the top 32 metabolites for all three comparisons.

**Table 2:** Metabolites that differed significantly between control and UQ groups at 2-y follow-up (*p*<0.05).

Metabolite	Molecular mass	p-value	Ratio
	(monoisotopic mass)		(UQ∶glycemic control)
**PHOSPHOLIPIDS**			
PC(34∶0)	759.5778	0.0092	0.81 (0.70, 0.94)
PC(34∶3)	755.5465	0.0114	0.75 (0.57, 0.97)
PG(40∶4)	826.5724	0.0144	0.92 (0.86, 0.98)
PC(18∶1/dm18∶1) AND/OR PC(18∶2/dm18∶0)	769.5985	0.0162	0.84 (0.71, 0.99)
PC(16∶0/dm18∶1) AND/OR PC(16∶1/dm18∶0) AND/OR PC(18∶1/dm16∶0)	743.5829	0.0226	0.81 (0.68, 0.98)
PS(20∶0)	567.3172	0.0304	0.84 (0.73, 0.97)
PC(32∶3)	727.5152	0.0317	0.81 (0.70, 0.95)
PC(18∶0)	509.3845	0.0323	0.80 (0.67, 0.97)
PC(31∶1)	717.5309	0.0339	0.86 (0.77, 0.97)
PE(42∶2)	827.6404	0.0363	0.88 (0.78, 0.98)
PC(16∶0/dm18∶1) AND/OR PC(16∶1/dm18∶0) AND/OR PC(18∶1/dm16∶0) AND/OR PC(34∶2)	743.5829	0.0406	0.85 (0.74, 0.98)
PC(16∶0)	481.3532	0.0485	0.86 (0.75, 0.99)
**LONG CHAIN FATTY ACIDS AND RELATED METABOLITES**			
Arachidonic acid *	304.2402	0.0128	0.86 (0.75, 0.98)
Hydroxy-dodecanoic acid	216.1725	0.0281	0.91 (0.82, 1.01)
N-(3-(hexadecanoyloxy)-heptadecanoyl)-ornithine	638.5598	0.0309	0.83 (0.71, 0.98)
Hydroxy-dodecadienoic acid AND/OR Oxo-dodecenoic acid	212.1412	0.0327	0.91 (0.78, 1.04)
Octadecadienoic acid *	280.2402	0.0385	0.85 (0.72, 1.01)
1,11-Undecanedicarboxylic acid AND/OR Methyl-dodecanedioic acid	244.1675	0.0496	0.91 (0.82, 1.01)
11-deoxy-PGE_1_ AND/OR 11-deoxy-PGF_2_ AND/OR 15-hydroperoxyeicosatrienoic acid	338.2457	0.0495	1.41 (0.88, 2.35)
**SHORT CHAIN FATTY ACIDS AND RELATED METABOLITES**			
Glyoxylic acid *	73.9993	0.0307	0.92 (0.86, 0.99)
Dimethylmalic acid AND/OR Ethylmalic acid AND/OR Hydroxyadipic acid	162.0528	0.0056	1.05 (0.97, 1.12)
Dimethylbutenoic acid	114.0681	0.0160	1.04 (0.95, 1.14)
2-Methylmaleic acid AND/OR Acetylpyruvic acid AND/OR Glutaconic acid AND/OR			
Itaconic acid AND/OR Mesaconic acid	130.0266	0.0249	1.03 (0.99, 1.08)
Methylacetoacetic acid	116.0473	0.0355	1.03 (0.97, 1.09)
2-Butenoic acid^a^ AND/OR Amino-methylpropanoic acid^b^ AND/OR Aminobutanoic acid^b^	86.03678^a^;103.063329^b^	0.0376	1.18 (0.96, 1.43)
**ACETYLCARNITINES**			
Dodecanoylcarnitine	343.2723	0.0123	0.75 (0.60, 0.93)
Octanoylcarnitine	287.2097	0.0200	0.79 (0.64, 0.97)
Decanoylcarnitine	315.2410	0.0210	0.77 (0.62, 0.95)
Tetradecanoyl carnitine	369.2879	0.0388	0.77 (0.60, 0.98)
**DIGLYCERIDES**			
DG(35∶0)	610.5536	0.0232	0.85 (0.74, 0.98)
DG(34∶0)	596.5380	0.0454	0.92 (0.85, 1.00)
DG(33∶0)	582.5223	0.0474	0.90 (0.81, 0.99)
DG(40∶3)	674.5849	0.0496	0.84 (0.72, 0.99)
**BILE ACIDS**			
24-Nor-5beta-cholane-3alpha,12alpha,22,23-tetrol AND/OR isomer	380.2927	0.0259	0.79 (0.65, 0.96)
**STEROIDS, PROSTANOIDS AND RELATED METABOLITES**			
Pregnane AND/OR			
2alpha-(Hydroxymethyl)-17-methyl-5alpha-androstane-3beta,17beta-diol			
AND/OR 5beta-Pregnane-3alpha,17alpha,20alpha-triol	336.2664	0.0066	0.78 (0.64, 0.95)
Leukotriene C_5_	623.2866	0.0340	0.86 (0.74, 1.00)
3beta-Hydroxyandrost-5-en-17-one 3-			
sulfate^a^ AND/OR 15-Hydroxy-5,8,11-cis-13-trans- eicosatetraenoic acid^b^	368.165746^a^;320.235145^b^	0.0172	1.36 (1.08, 1.70)
**AMINO ACIDS AND RELATED METABOLITES**			
Proline Betaine	143.0946	0.0181	0.94 (0.89, 0.99)
Urea *	60.03240	0.0388	0.92 (0.82, 1.03)
Thiourocanic acid	170.0150	0.0408	0.93 (0.87, 1.00)
**OTHERS**			
3-Deoxyvitamin D_3_	368.3443	0.0309	0.81 (0.67, 0.97)
Enterostatin (VPGPR)	524.3071	0.0405	0.78 (0.62, 1.00)
Decanol	158.1671	0.0454	1.03 (0.99, 1.07)

Metabolites have been classified into structural or functional classes. Within each class, data have been separated into those with higher (where such exist) and lower ratios, respectively, and are then presented in order from lowest to highest *p* value. The molecular weights, calculated as the monoisotopic mass, are included. Ratios with 95% confidence intervals in parentheses are shown. DM, demethyl, G, glycine; P, proline; PC, phosphatidylcholine; PE, phosphatidylethanolamine; PG, glycerophosphoglycerol; PS, phosphatidylserine; R, arginine; V, valine; The values in parentheses (for example PC(34∶0)) relate to the total fatty acid carbon chain length and number of carbon double bonds (unsaturation) in each metabolite. *Identification by matching of retention time and accurate mass to authentic chemical standard

**Table 3:** Metabolites differing between control and GDM groups at 2-y follow-up (*p*<0.05).

Metabolite	Molecular mass	p-value	Ratio
	(monoisotopic mass)		(GDM∶glycemic control)
**PHOSPHOLIPIDS**			
LysoPC(10∶1)	395.2437	0.0114	0.79 (0.58, 1.01)
PI(36∶3)	860.5415	0.0188	0.90 (0.83, 0.97)
PC(34∶0)	747.6142	0.0206	0.81 (0.69, 0.94)
PC(38∶0)	817.6561	0.0328	0.80 (0.66, 0.96)
LysoPC(16∶2)	477.3219	0.0481	0.88 (0.77, 1.01)
LysoPE(18∶1)	479.3012	0.0489	0.83 (0.69, 0.99)
LysoPC(20∶1)	549.3794	0.0430	1.03 (1.00, 1.06)
LysoPG(18:1)	510.2958	0.0499	1.04 (0.86, 1.26)
PS(37∶0)	805.5833	0.0291	1.09 (1.01, 1.16)
**LONG CHAIN FATTY ACIDS AND RELATED METABOLITES**			
Tetradecenoic acid *	226.1933	0.0142	0.91 (0.86, 0.97)
Decadiynoic acid	164.0837	0.0203	0.58 (0.40, 0.85)
Hydroxydodecanoic acid	216.1725	0.0303	0.71 (0.56, 0.90)
Hydroxyoctadecanoic acid	300.2664	0.0312	0.74 (0.63, 0.89)
Docosanol	326.3549	0.0394	0.79 (0.66, 0.95)
3-Isopropenyl-6-oxoheptanoic acid AND/OR Oxodecenoic acid	184.1099	0.0073	1.38 (1.05, 1.80)
Decanol	158.1671	0.0109	1.07 (1.00, 1.13)
Nonadienoic acid	154.0994	0.0181	1.11 (1.03, 1.20)
Elaidoylamide	281.2719	0.0202	1.09 (0.96, 1.24)
Octenedioic acid AND/OR Dioxo-octanoic acid	172.0736	0.0250	1.21 (0.93, 1.49)
Hydroxydodecanoic acid	216.1725	0.0487	1.23 (0.89, 1.65)
**SHORT CHAIN FATTY ACIDS AND RELATED METABOLITES**			
Hydroxypyruvic acid AND/OR Malonic acid	104.0110	0.0029	0.92 (0.86, 0.97)
Acetic acid^a^ AND/OR Glyceric acid^b^	60.02113^a^;106.02661^b^	0.0114	0.92 (0.87, 0.98)
Methylmalonic acid semialdehyde AND/OR Methyloxopropanoic acid	102.0317	0.0125	0.93 (0.88, 0.98)
Propanoic acid^a^ AND/OR Dihydroxybutyric acid^b^ AND/OR Deoxyerythronic acid^b^	74.03678^a^;120.04226^b^	0.0237	0.93 (0.87, 0.99)
2-Amino-3-phosphonopropanoic acid^a^ AND/OR 2-hydroxysuccinamic acid^b^	169.014011^a^;133.037509^b^	0.0254	0.62 (0.44, 0.89)
Methylvaleric acid AND/OR Dimethylbutanoic acid	116.0837	0.0470	0.82 (0.66, 0.99)
Dimethylmalic acid AND/OR Ethylmalic acid AND/OR Hydroxyadipic acid	162.0528	0.0024	1.11 (1.02, 1.22)
8-Amino-7-oxononanoate	187.1208	0.0049	1.49 (1.17, 1.98)
Butenoic acid *			
AND/OR Oxobutanoic acid AND/OR Methylpyruvic acid	84.0211	0.0079	1.16 (1.04, 1.28)
Ethylhexenoic acid AND/OR Methylheptenoic acid AND/OR Octenoic acid	142.0994	0.0139	1.25 (1.06, 1.48)
Oxopentanoic acid AND/OR Methyloxobutanoic acid	116.0473	0.0241	1.21 (1.01, 1.42)
Oxo-hydroxy-aminovaleric acid	147.0532	0.0350	1.32 (1.03, 1.65)
Methylmaleic acid AND/OR Acetylpyruvic acid AND/OR			
Itaconic acid AND/OR Mesaconic acid	130.0266	0.0445	1.03 (0.99, 1.08)
Hydroxyhexanoic acid AND/OR Ethyl-Hydroxybutyric acid AND/OR			
Hydroxymethylpentanoic acid	132.0786	0.0481	1.18 (0.93, 1.48)
**DIGLYCERIDES**			
DG(40∶1)	678.6162	0.0167	0.78 (0.66, 0.93)
**BILE ACIDS**			
Chenodeoxycholic acid 3-sulphate	472.2495	0.0279	0.87 (0.76, 1.00)
3alpha,12alpha-Dihydroxy-5beta-chol-22-en-24-oic Acid			
AND/OR isomers	390.2770	0.0481	0.94 (0.87, 1.00)
5beta-Cholane-3alpha-24-diol	410.3185	0.0108	1.10 (1.02, 1.18)
**STEROIDS, PROSTANOIDS AND RELATED METABOLITES**			
4alpha,24beta-Dimethyl-5alpha-cholest-22-en-3beta-4beta-diol AND/OR			
4alpha-hydroxymethyl-4beta-methyl-5alpha-cholesta-8-en-3beta-ol			
AND/OR isomers	430.3811	0.0131	0.78 (0.63, 0.95)
25-Azacholesterol	387.3501	0.0499	0.93 (0.83, 1.05)
PGF2alpha-11-acetate	396.2512	0.0025	1.11 (1.03, 1.19)
11alpha-hydroxy-9,15-dioxoprost-13-enoate	352.2250	0.0054	1.12 (1.02, 1.24)
3alpha,11beta,17alpha-Trihydroxy-5beta-pregnan-20-one AND/OR isomers	350.2457	0.0499	1.07 (0.99, 1.15)
**AMINO ACIDS AND RELATED METABOLITES**			
N-(Aminomethyl)urea	89.0589	0.0045	0.52 (0.35, 0.80)
Phosphoshikimate	254.0192	0.0104	0.85 (0.76, 0.95)
Tryptophan *	204.0899	0.0139	0.90 (0.83, 0.98)
Uric acid *	168.0283	0.0177	1.11 (1.02, 1.20)
Proline *	115.0633	0.0185	1.09 (0.90, 1.36)
Leucine AND/OR Isoleucine AND/OR Norleucine AND/OR N-methylvaline	131.0946	0.0268	1.27 (1.02, 1.54)
Dimethyluric acid	196.0596	0.0365	1.17 (1.01, 1.36)
**UBIQUINONE AND STEROL BIOSYNTHESIS AND RELATED METABOLITES**			
2-Polyprenylphenol	230.1671	0.0107	0.69 (0.44, 1.02)
Benzosemiquinone	110.0368	0.0346	0.56 (0.38, 0.88)
2-Hexaprenylphenol	502.4175	0.0368	0.89 (0.81, 0.97)
**TETRAHYDROFOLATE METABOLISM**			
5,6,7,8-Tetrahydrofolate	445.1710	0.0206	0.84 (0.68, 1.01)
5-Methyltetrahydropteroyltriglutamate	717.2718	0.0041	1.21 (1.00, 1.46)
**OTHERS**			
Glucosylceramide(42∶1)	811.6901	0.0051	0.81 (0.68, 0.96)
Pantetheine 4'-phosphate^a^ AND/OR N-((R)-Pantothenoyl)-L-cysteine^b^	358.096361^a^;322.119859^b^	0.0079	0.94 (0.90, 0.97)
Teasterone AND/OR Typhasterol	448.3553	0.0279	0.80 (0.65, 0.95)
Methylguanosine	297.1073	0.0369	0.91 (0.85, 0.98)
Methylglucuronic Acid^a^ AND/OR Dihydrolipoic acid^b^	208.058305^a^;208.059172^b^	0.0006	1.13 (1.02, 1.25)
Propane-1,2-diol-1-phosphate^a^ AND/OR 2-Hydroxy-2-methylbutyric acid^b^	156.018762^a^;118.062995^b^	0.0034	1.59 (1.13, 2.18)
Thiourocanic acid	170.0150	0.0042	1.16 (1.05, 1.27)
Nonylglucoside	306.2042	0.0070	1.61 (1.05, 2.24)
CDP-4-dehydro-6-deoxy-D-glucose	547.0604	0.0108	1.10 (1.01, 1.21)
Monosaccharide	180.0634	0.0136	1.14 (1.03, 1.27)
5,7,22,24(28)-ergostatetraenol^a^ AND/OR			
20-cyclopropyl-1alpha,25-dihydroxy-16,17-didehydro-21-norvitamin D_3_^b^	394.323565^a^;440.329045^b^	0.0185	1.38 (1.02, 1.81)
Ribose 1,5-bisphosphate AND/OR isomer	309.9855	0.0365	1.13 (1.00, 1.27)
CE(15∶0)	610.5689	0.0400	1.27 (0.95, 1.66)

Metabolites have been classified according to their molecular structures or known metabolic functions/pathway participation. Within each class the data have been separated in to those with higher and lower ratios and are then presented in order from lowest to highest *p* value. The molecular weights, calculated as the monoisotopic mass, are included. Ratios with 95% confidence intervals in parentheses are shown. CE cholesteryl ester; DG, diglyceride; PC, phosphatidylcholine; PE, phosphatidylethanolamine; PG, phosphatidylglycine; PGF, prostaglandin; PI, phosphatidylinositol; PS, phosphatidylserine; The values in parentheses (for example PC(34∶0)) relate to the total fatty acid carbon chain length and number of carbon double bonds (unsaturation) in each metabolite. *Identification by matching of retention time and accurate mass to authentic chemical standard.

**Table 4:** Metabolites differing between UQ and GDM groups at 2-y follow-up (*p*<0.05).

Metabolite	Molecular mass	p-value	Ratio
	(monoisotopic mass)		(GDM∶UQ)
**PHOSPHOLIPIDS**			
LysoPC(16∶2)	477.3219	0.0196	0.88 (0.77, 0.99)
PC(36∶3)	767.5829	0.0378	0.91 (0.83, 0.99)
PG(40∶5)	824.5567	0.0056	1.17 (1.01, 1.35)
PC(48∶1)	759.5778	0.0099	1.19 (1.00, 1.40)
LysoPC(18∶0)	523.3638	0.0214	1.11 (0.97, 1.27)
PC(19∶0)	537.3794	0.0234	1.31 (1.00, 1.67)
PC(32∶3)	727.5152	0.0287	1.16 (1.01, 1.32)
LysoPC(16∶0)	495.3325	0.0292	1.07 (0.99, 1.16)
PC(14∶1/dm16∶0)^a^ AND/OR PE(15∶0/dm18∶1)^b^ AND/OR PC(34∶6)^b^	687.520291^a^;749.499556^b^	0.0301	1.13 (1.01, 1.27)
PC(36∶1)	787.6091	0.0347	1.13 (0.98, 1.31)
PC(17∶0)	509.3481	0.0411	1.25 (1.00, 1.55)
**LONG CHAIN FATTY ACIDS AND RELATED METABOLITES**			
Tetradecenoic acid *	226.1933	0.0037	0.90 (0.84, 0.96)
Eicosanol	298.3236	0.0305	0.87 (0.80, 0.96)
Dimethylundecanoic acid AND/OR Methyldodecanoic acid	214.1933	0.0445	0.86 (0.74, 0.99)
Tridecadienoic acid	210.1620	0.0045	1.21 (1.06, 1.38)
Octadecadienol	266.2610	0.0047	1.20 (1.06, 1.36)
Nonadienoic acid	154.0994	0.0134	1.81 (1.28, 2.52)
Elaidoylamide	281.2719	0.0215	1.12 (0.97, 1.28)
Hydroxydodecadienoic acid AND/OR Oxododecenoic acid	212.1412	0.0256	1.08 (1.01, 1.15)
Nonadienoic acid *	154.0994	0.0269	1.11 (1.02, 1.19)
Hexacosatrienoic acid *	390.3498	0.0419	1.66 (0.98, 2.46)
Hydroxydecanoic acid	188.1412	0.0446	1.23 (0.95, 1.54)
**SHORT CHAIN FATTY ACIDS AND RELATED METABOLITES**			
Hydroxypyruvic acid AND/OR Malonic acid	104.0110	0.0035	0.91 (0.86, 0.97)
Acetic acid AND/OR Glyceric acid	60.02113^a^;106.02661^b^	0.0205	0.93 (0.87, 0.98)
Methylmalonic acid semialdehyde AND/OR Methyl-oxopropanoic acid			
AND/OR Oxobutanoic acid AND/OR Methylpyruvic acid	102.0317	0.0280	0.93 (0.88, 0.99)
Methylvaleric acid AND/OR Dimethylbutanoic acid	116.0837	0.0329	0.81 (0.66, 0.98)
Propanoic acid^a^ AND/OR Dihydroxybutyric acid^b^ AND/OR Deoxyerythronic acid^b^	74.03678^a^;120.04226^b^	0.0362	0.93 (0.86, 0.99)
Diaminopropanoic acid^a^ AND/OR Hydroxy-oxoglutaric acid^b^	104.058578^a^;162.01644^b^	0.0378	0.93 (0.87, 1.00)
Hydroxyheptynoic acid	142.0630	0.0484	0.91 (0.83, 1.00)
Decadiynoic acid	164.0837	0.0498	0.70 (0.50, 0.97)
Isopropenyl-oxoheptanoic acid AND/OR Oxodecenoic acid	184.1099	0.0066	1.26 (0.86, 2.09)
Hydroxyheptanoic acid	146.0943	0.0181	1.14 (1.01, 1.28)
Butynoic acid	84.0211	0.0268	1.11 (1.00, 1.22)
Amino-oxononanoic acid	187.1208	0.0287	1.29 (1.00, 1.71)
Oxopentanoic acid AND/OR Methyl-oxobutanoic acid	116.0473	0.0413	1.17 (0.98, 1.38)
Octenedioic acid AND/OR Dioxo-octanoic acid	172.0736	0.0480	1.15 (0.88, 1.45)
**DIGLYCERIDES**			
DG(32∶0)	568.5067	0.0319	1.13 (1.01, 1.25)
DG(34∶0)	624.5693	0.0333	1.15 (1.01, 1.30)
DG(43∶3)	718.6475	0.0378	1.08 (0.97, 1.20)
DG(36∶1)	610.5536	0.0481	1.23 (0.98, 1.50)
**BILE ACIDS**			
Chenodeoxycholic acid 3-sulfate	472.2495	0.0347	0.88 (0.76, 1.01)
3alpha,12alpha-Dihydroxy-5beta-chol-22-en-24-oic Acid AND/OR isomers	390.2770	0.0446	0.91 (0.84, 1.00)
Glycochenodeoxycholic acid 3-glucuronide	625.3462	0.0484	0.49 (0.23, 1.03)
5-beta-Cholane-3alpha,24-diol	410.3185	0.0002	1.16 (1.08, 1.24)
**STEROIDS, PROSTANOIDS AND RELATED METABOLITES**			
4alpha,24beta-Dimethyl-5alpha-cholest-22-en-3beta-4beta-diol AND/OR			
4alpha-hydroxymethyl-4beta-methyl-5alpha-cholesta-8-en-3beta-ol AND/OR isomers	430.3811	0.0051	0.75 (0.60, 0.91)
25-Azacholesterol	387.3501	0.0428	0.95 (0.85, 1.06)
Leukotriene C_5_	623.2866	0.0032	1.28 (1.10, 1.50)
11alpha-Hydroxy-9,15-dioxoprost-13-enoate	352.2250	0.0035	1.18 (1.06, 1.30)
PGF2alpha-11-acetate	396.2512	0.0095	1.12 (1.03, 1.23)
11beta-Hydroxyprogesterone AND/OR 17beta-Hydroxy-4-oxa-5alpha-androst-1-en-3-one acetate			
AND/OR 5,6-epoxy,18R-HEPE	332.1988	0.0160	1.24 (0.90, 1.72)
**AMINO ACIDS AND RELATED METABOLITES**			
N-(Aminomethyl)urea	89.0589	0.0023	0.60 (0.44, 0.83)
Phosphoshikimate	254.0192	0.0116	0.84 (0.74, 0.95)
Hydantoin	100.0273	0.0428	0.96 (0.92, 1.00)
Tryptophan *	204.0899	0.0171	0.90 (0.82, 0.98)
Methylcrotonylglycine AND/OR Tiglylglycine	157.0739	0.0448	0.51 (0.28, 0.93)
Proline *	115.0633	0.0048	1.10 (1.03, 1.19)
2-Oxoglutaramate	145.0375	0.0154	2.11 (0.79, 3.96)
Leucine OR Isoleucine AND/OR Norleucine AND/OR N-methylvaline	131.0946	0.0464	1.21 (0.96, 1.50)
**UBIQUINONE AND STEROL BIOSYNTHESIS AND RELATED METABOLITES**			
2-Hexaprenylphenol	502.4175	0.0105	0.88 (0.81, 0.97)
2-Polyprenylphenol	230.1671	0.0220	0.72 (0.46, 1.05)
5-Phosphomevalonate	228.0399	0.0245	0.92 (0.84, 1.00)
Benzosemiquinone	110.0368	0.0464	0.67 (0.48, 0.93)
2-trans,6-trans-Farnesal OR 4-n-Nonylphenol	220.1827	0.0446	1.23 (0.93, 1.55)
**TETRAHYDROFOLATE METABOLISM**			
5,6,7,8-Tetrahydrofolate	445.1710	0.0131	0.86 (0.70, 1.02)
**OTHERS**			
Hydroxycholesterol AND/OR 12,14-Heptacosadiynoic acid AND/OR			
12alpha-Hydroxy-5beta-cholestan-3-one AND/OR 19-Hydroxy-10S,19-dihydrovitamin D3	402.3498	0.0111	0.77 (0.64, 0.93)
Pantetheine 4'-phosphate^a^ AND/OR N-((R)-Pantothenoyl)-L-cysteine^b^	358.096361^a^;322.119859^b^	0.0292	0.95 (0.92, 0.99)
CE(16∶2)	620.5532	0.0464	0.93 (0.80, 1.07)
CE(15∶0)	610.5689	0.0061	1.28 (0.94, 1.73)
CDP-4-dehydro-6-deoxy-D-glucose	547.0604	0.0070	1.12 (1.02, 1.22)
14-methyl-20,14-retro-retinoic acid AND/OR 16,17-Didehydropregnenolone AND/OR			
17beta-Hydroxy-2alpha,17-dimethyl-4,9(11)-androstadien-3-one AND/OR isomers	314.2246	0.0179	1.28 (0.93, 1.77)
Hexose sugar	180.0634	0.0214	1.12 (1.01, 1.24)
Alpha-CEHC-glucuronide	454.1839	0.0458	1.10 (0.99, 1.20)

Metabolites have been classified according to their molecular structures or known metabolic functions/pathway participation. Within each class, data have been separated in to those with higher and lower ratios and are then presented in order from lowest to highest *p* value. The molecular weights, calculated as the monoisotopic mass, are included. Ratios with 95% confidence intervals in parentheses are shown. CE Cholesteryl ester; CEHC, 2,5,7,8-tetramethyl-2-(2'-carboxyethyl)-6-hydroxychroman; DG, diglyceride; HEPE, hydroxy-eicosapentaenoic acid; PC, phosphatidylcholine; PG, phosphatidylglycine; The values in parentheses (for example PC(34∶0)) relate to the total fatty acid carbon chain length and number of carbon double bonds (unsaturation) in each metabolite. *Identification by matching of retention time and accurate mass to authentic chemical standard.

**Figure 3:**
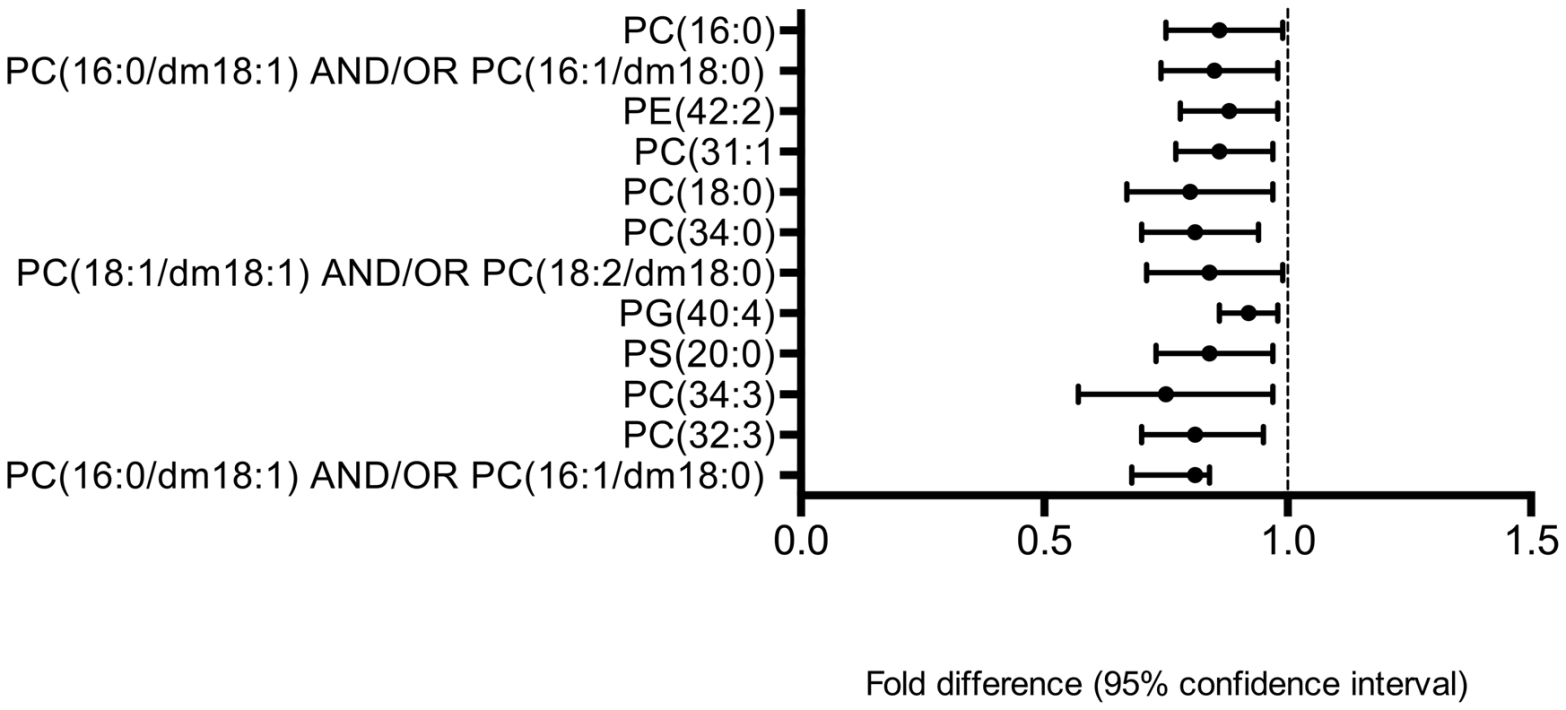
Phospholipids that differed significantly between control and UQ groups at 2-y follow-up (*p*<0.05). *Identification by matching of retention time and accurate mass to authentic chemical standard.

**Figure 4:**
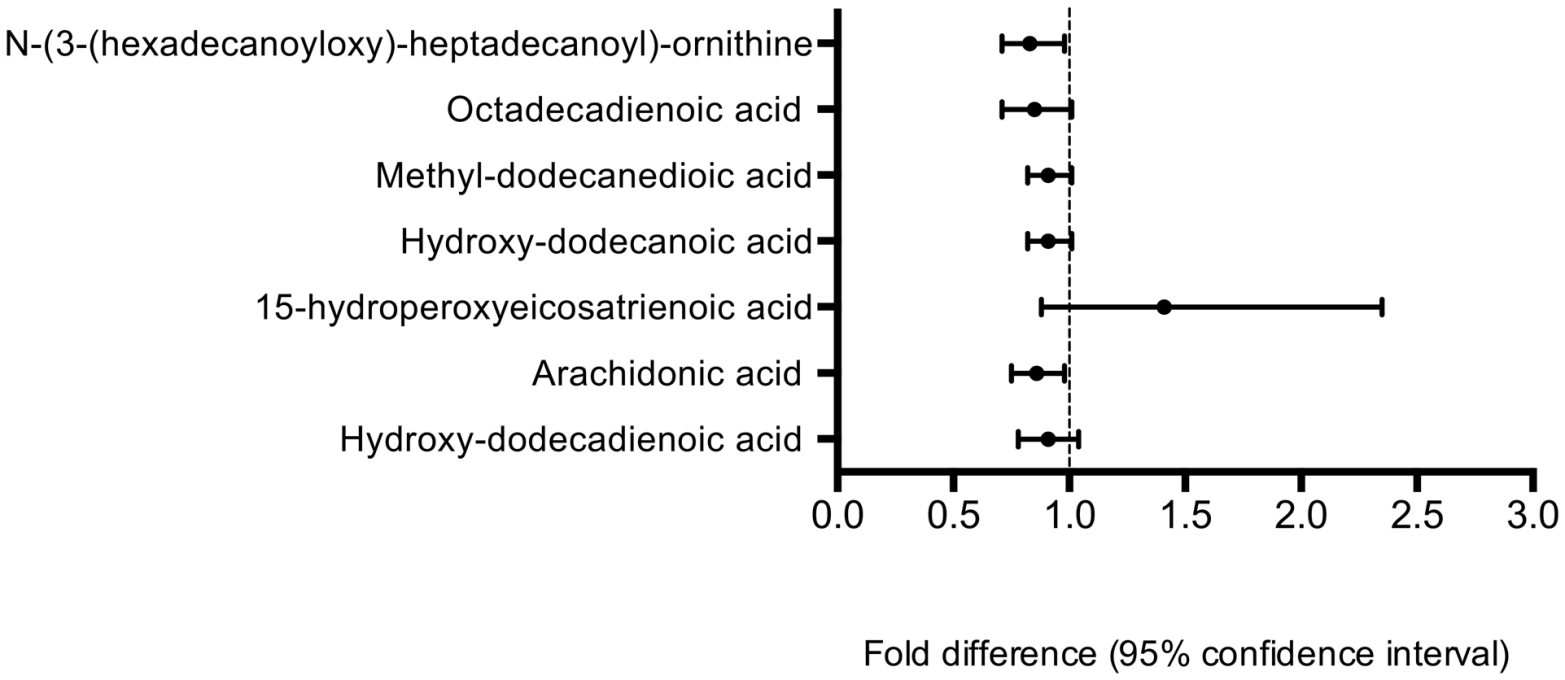
Long chain fatty acids that differed significantly between control and UQ groups at 2-y follow-up (*p*<0.05). PC, phosphatidylcholine; PE, phosphatidylethanolamine; PG, glycerophosphoglycerol; PS, phosphatidylserine.

By comparing the control and UQ groups, 173 of 3552 metabolic features were statistically different (p<0.05). Of these, 43 unique metabolic features were annotated (Table 2). 35 metabolites, notably those classified in the phospholipid (Figure 3) and long-chain fatty acid classes (Figure 4), were present at lower concentrations in the UQ than in controls, as were levels of certain vitamin D metabolites and the anorectic pentapeptide, enterostatin [52].

In comparisons between the Control and GDM groups, 392 of 3,552 metabolic features differed significantly. Of these 392 metabolites, 69 *unique* metabolic features were annotated (Table 3). Here the picture was more evenly balanced, with about one half of the metabolites in each class higher (35 of 69 metabolites) and the remainder lower in the UQ than the control group.

For the UQ versus GDM comparison, 401 of 3552 metabolic features differed significantly, of these, 72 *unique* metabolic features were annotated, (Table 4). Many of the metabolites annotated in Table 3 recurred in Table 4, but notably the metabolite 2-Oxoglutaramate, an important biomarker of hepatic encephalopathy and other hyperammonemic diseases [53], [54] was twice as abundant in previous GDM compared those in the UQ subgroup (2.11 (0.79, 3.96; p = 0.015)). A notable fold change in the metabolite N-(aminomethyl)urea was observed between the groups (Table 3 and Table 4).

## Discussion

The pathophysiological metabolic changes in the very early stages of type 2 diabetes, before measurable hyperglycemia, remain comparatively little known or understood. Our current results provide compelling evidence for the occurrence of significant metabolic defects that antedate the onset of hyperglycemia, even if marginal differences in glycemia well within the normal range were present. These metabolic defects may exert effects that can lead to or cause subsequent glucoregulatory decompensation deteriorating to ‘hyperglycemia’, which currently defines the disease.

The particular metabolic pathways suggested by this study are defects in those regulating systemic lipid metabolism [55] and hormone secretion/responsiveness [56]; they appear to antedate and could therefore ‘cause’ or lead to overt hyperglycemia. Hormones currently implicated in the development of T2DM include the beta-cell hormones insulin and amylin [55], [56], and the adipocyte hormones leptin [57] and adiponectin [58]. Early damage to blood vessels [1], [59] and pancreatic islet beta-cells [60], for example, provide evidence for metabolic defects that antedate diabetes. Copper homeostasis and iron status are also related to GDM [61]–[63]. For example, high body iron stores, leading to unliganded iron, cause hydroxyl radical formation via Fenton chemistry and are significantly associated with a greater risk of T2DM [22], [64]–[67]. Here, 29 of the women were included in a vascular sub-study where there was a gradation of declining endothelial function of resistance blood vessels ex-vivo, poorest in the 12 of the 18 women with prior GDM studied here and less marked in those with UQ, compared with controls defined the same way [3]. Those vascular findings parallel the metabolic changes reported here.

To address questions of what metabolic markers identify the pathogenic pathways to T2DM and from them potential new strategies for disease prevention, we compared the 2 at-risk groups with controls to quantify specific metabolic differences between groups. The data suggest that some pathogenic processes may have begun by the time women reached the UQ state, with others underway when they further deteriorate, previously indicated by being GDM. Several distinct if overlapping molecular processes may underpin these successive degrees of regulatory impairment represented by the two increased-risk states. Dividing the complex time-dependent process into stages produces artificial categories but enables identification of earlier- and later-onset pathways.

Twenty-two months after their index pregnancy, when originally profiled by their glucose tolerance, the women had this status re-assessed by fasting plasma glucose and hemoglobin A_1c_ values. In contrast to their within-pregnancy glucose tolerance, glycaemic indices at re-testing were not different between the UQ and GDM groups, although both were marginally defective compared to controls, yet still within the usual, ‘currently normal’ glycaemic range. Pair-wise between-group comparisons pinpointed relatively circumscribed subsets of defined metabolite classes related to elevated diabetes risk. Those metabolite classes perturbed in the UQ compared with control women included: phospholipid subclasses, in particular phosphatidylcholines; LCFA; LCFA-carnitines; SCFA and SCFA-metabolites. Other perturbed classes included diglycerides; bile acids; steroids; prostanoids; and amino acid metabolites. Most of these belong to lipid sub-classes. The greatest differences here were in the acyl carnitine class.

Prominent differences in phosphatidylcholines were identified in both the control/UQ and UQ/GDM contrasts. Diacyl-phosphatidylcholines has been shown to be independently associated with increased risk of type 2 diabetes in a prospective study of type 2 diabetes patients in the European Prospective Investigation into Cancer and Nutrition (EPIC)-Potsdam cohort [27]. Phospholipids are highly insoluble in aqueous media so these molecules will have originated in membranous structures in plasma, namely lipoproteins; this suggests that differences in phosphatidylcholine composition are related and could contribute to glucoregulatory transitions preceding hyperglycemia. Alterations in additional lipid classes including those of steroids/bile acids, and diglycerides are also probably related to changes in lipoprotein metabolism. Consistent with these findings, diabetes itself is associated with prominent changes in plasma lipoprotein content [68], [69]. This disturbance in phospholipid metabolism cannot be localised or characterised further here since the observed changes could reflect alterations in any or all of the HDL, LDL, or VLDL fractions. Prominent alterations in LDL-particle composition have previously been identified in diabetes pathogenesis [70], lipoprotein-bound phospholipids are reportedly targets of glycoxidation-mediated damage [71], and oxidized phospholipids can become pathogenic [72], [73]. Such direct effects of lipid alterations on blood vessels possibly underlie the major benefits of statin treatment in T2DM, although statins are also thought to be anti-inflammatory [22], [74]. Whether, and through what pathways, statins may lead to increases in glycemia [17], [18] remain unanswered questions relevant to this early pathogenesis. Altered lipoproteins are also implicated in the mechanisms that lead to or cause beta-cell dysfunction in diabetes [75]. Follow-up proteomic and metabolomic studies of purified lipoprotein fractions from different classes of at-risk patients would now help identify the specific molecules more clearly and may in time be useful in improving the performance of classification models based on standard factors [76]. The data here clearly point to early alteration in lipoprotein metabolism in the chain of events that culminate in diabetes and its complications.

A lysophospholipid-related signal may also be present, particularly in the UQ/GDM and control/GDM comparisons, indicating the onset of pro-inflammatory stress, which contributes to tissue damage. Plasma lyosphosphospholipid content is another potential biomarker for monitoring oxidative damage caused, for example [77] by perturbed regulation of catalytically-active copper metabolism before and in diabetes [78]. Lysophospholipid measurements could help monitor progression of tissue damage in people at risk of developing diabetes, and perhaps the response to preventive/therapeutic interventions.

Another significantly perturbed lipid-related signal here was for LCFA and LCFA-carnitines. Both classes tended to be lower in the UQ than in controls. Gall et al reported that medium-chain acylcarnitines such as decanoylcarnitine decreased in concentration with increasing insulin resistance and dysglycemia [35]. In the population-based Cooperative Health Research in the Region of Augsburg (KORA) cohort, three metabolites, namely glycine, lysophosphatidylcholine (LPC) (18∶2) and acetylcarnitine had significantly altered levels in IGT individuals as compared to those with normal glucose tolerance [79]. Acylcarnitines are biosynthesized solely in mitochondria, where they transport fatty acids into the organelle for beta-oxidation, so decreases in their plasma levels might reflect increased mitochondrial utilisation [80]. Here, serum levels of both LCFA and LCFA-carnitines were lower in UQ compared to control women, consistent with increased rates of tissue fatty acid utilisation in the UQ group. Such changes can occur in the glucose-sparing fuel economy that emerges in diabetes [81]. Preferential fatty-acid utilisation may contribute to systemic hyperglycemia as recognised long ago [82]. Our data indicate that such utilisation begins much earlier in the pathogenic process than hitherto recognised. The lowering of LCFA and LCFA-carnitines coincided with a small increase in fasting plasma glucose in the UQ group, consistent with substitution of LCFA for glucose in mitochondrial oxidation. Perturbations in LCFA metabolism have been implicated in the pathogenesis of beta-cell damage in diabetes [83]; the early onset of altered LCFA here may lead to or cause beta-cell dysfunction/damage [57]. Acyl carnitine levels were elevated in pregnant women who went on to develop pre-eclampsia [34]. By contrast, this pattern is no longer evident in the UQ/GDM comparison, where LCFA tended to be higher, probably consistent with their impaired mitochondrial oxidation, typical of insulin resistance in the former (and fatter) GDM group.

Another complex metabolic alteration change more prominent in the UQ/GDM comparison is a tendency to increased numbers of SCFA and SCFA-metabolites. Elevated SCFA and SCFA-metabolites suggest their defective utilisation, as in diabetes [81], again occurring earlier than hitherto realised. Shikimate 3-phosphate an obligatory intermediate in the anabolic pathway for biosynthesis of the essential aromatic amino acids, is potentially a microbial metabolite not produced in human cellular metabolism [84]. Some SCFA-metabolites identified may originate from microbial biosynthesis. The identification of microbial metabolites in human plasma with possible links to defective glucoregulation could point to between-group differences in their production by gut microflora and/or uptake from the gut. Other identified metabolic features, as in terpenoid/quinones and teasterone/typhasterol may be of plant origin, consistent with possible differences in dietary intake and/or uptake from the gut.

We also found (Table 1) that significantly lower circulating adiponectin levels occurred before measurable alterations in insulin or leptin levels. Adiponectin deficiency occurs from infancy, as found in the children of this cohort [85] and may influence GDM [28] and T2DM [86]–[88]. It is associated with defective glycosylation and functionality, such as impaired ability to stimulate hepatic or muscle mitochondrial fatty acid oxidation via AMP kinase [58], [89]. Adiponectin deficiency could provide a central link between perturbed phosphatidylcholine metabolism and mitochondrial lipid utilisation here. However, whether changes in production/secretion and/or signalling of known hormones including adiponectin really antedate or rather result from the described metabolic changes remains uncertain. It is certainly known that adiponectin deficiency can cause these changes but together with the exact nature and origin of the adiponectin deficiency observed here, requires further longitudinal study.

In summary, we identified here a rather consistent pattern of metabolic perturbations in groups of women whose diabetes risk was stratified *a priori* by differences in their degree of glucoregulatory impairment during a previous pregnancy. The data point to a time-line in the molecular pathologies ultimately leading to type 2 diabetes; the changes found in the control/UQ comparison likely precede those in the UQ/GDM comparison (e.g. perturbed plasma phospholipids and altered lipoprotein metabolism). A second early alteration was the relative fall in plasma LCFA and LCFA-carnitines, along with minor increases in fasting plasma glucose and HbA_1c_ levels. Those are consistent with a glucose-sparing mitochondrial fuel economy, related to the increased abdominal circumference in the UQ and GDM groups.

Many changes occurred in clusters of metabolite classes, for example phospholipids, lysophospholipids, LCFA, LCFA-carnitines, and SCFA/SCFA-metabolites, pointing to mechanisms that affect large subsets of these metabolite classes (e.g. transcription factors), long before the emergence of overt disease. Differences in relative timings of activation in different potential pathways to the onset/progression of T2DM pathogenesis were also observed. Modified lysophospholipid metabolism possibly implies elevated pro-inflammatory stress; lowered LCFA/LCFA-carnitine levels are consistent with early metabolic fuel substitution leading to preferential mitochondrial oxidation of LCFA as opposed to glucose, providing an early hyperglycemic stimulus; a widespread increase in SCFA/SCFA-metabolites suggest potential early defects in their generation and/or defective mitochondrial utilisation.

Finally, we found early adiponectin deficiency which may initiate or contribute to several of the metabolic disturbances, The results point to a probable defect in adipose tissue regulation contributing to the initiation of T2DM pathogenesis; further characterisation of the early changes in adiponectin synthesis and post-translational modifications and its causes will be useful. Our current conclusions are reminiscent in several respects of those from a recent study of the antecedents of type 1 diabetes wherein dysregulation of lipid and amino acid metabolism preceded islet autoimmunity in children who later progressed to overt disease [37].

Our study paves the way for targeted investigation of the pathogenic biochemical pathways that lead to or cause type 2 diabetes and more effective prevention and therapy [90], notably of blood vessel damage. Further longitudinal studies of diabetes development as we are doing here will be needed for assessing those at risk in general populations. Our study highlights the important role of metabolic profiling in discovery studies related to diabetes. Although metabolite identifications are not definitive they provide mechanistic information to guide further targeted studies. The major perturbations in this hypothesis-generating stage affected large subsets of metabolite classes showing co-variation between metabolites. Therefore, no corrections for multiple comparisons were applied. Finally, whether these patterns of metabolic derangements after prior GDM may lead to or cause the T2DM in general populations needs testing.
